# Association of Elevated Body Mass Index with Tibial Tuberosity Avulsion Fractures in Pediatric Athletes: A Pilot Retrospective Study

**DOI:** 10.3390/medicina61091698

**Published:** 2025-09-18

**Authors:** Josip Kocur, Slavko Čičak, Damjan Dimnjaković, Izabela Kiš, Gordana Kristek, Krešimir Ivković, Dalibor Kristek, Dalibor Divković

**Affiliations:** 1 Faculty of Medicine, Josip Juraj Strossmayer University of Osijek, J. Huttlera 4, 31000 Osijek, Croatiadivkovicdalibor1369@gmail.com (D.D.); 2 Department of Orthopaedics and Traumatology, University Hospital Centre Osijek, 31000 Osijek, Croatia; 3 Department of Orthopaedic Surgery, University Hospital Centre Zagreb, 10000 Zagreb, Croatia; 4 Department of Pediatric Surgery, University Hospital Centre Osijek, 31000 Osijek, Croatia; 5 Department of Anesthesiology, Resuscitation and ICU, University Hospital Centre Osijek, 31000 Osijek, Croatia

**Keywords:** tibial tuberosity fractures, pediatric injuries, adolescent, body mass index, knee fractures, overweight

## Abstract

*Background and Objectives*: Tibial tuberosity avulsion fractures are rare injuries in pediatric athletes, with limited data on the potential role of an elevated body mass index (BMI) as a risk factor. Previous studies have primarily focused on age, sex, and sport type, but the association between BMI and these injuries remains underexplored. Tibial tuberosity avulsion fractures are rare injuries predominantly affecting adolescent boys during sports activities involving strong quadriceps contractions. This pilot study aimed to analyze the epidemiological and anthropometric characteristics of patients with these fractures, including the distribution of injury mechanisms and the fracture types, to test whether the prevalence of overweight/obesity among cases exceeded national population benchmarks, and to describe the associated clinical outcomes. *Materials and Methods*: A retrospective analysis was conducted on medical records and radiographs of patients under the age of 18 treated between 2017 and 2024. The data collected included demographic and anthropometric characteristics, injury mechanisms, fracture classification, treatment methods, complications, and outcomes. The patients were categorized as normal weight (<85th percentile) or overweight/obese (≥85th percentile). The primary outcome was whether the prevalence of overweight/obesity among the cases exceeded national pediatric benchmarks. Formal sample size and power analyses were performed to guide future research. *Results*: Twenty-one patients met the inclusion criteria, with a mean age of 13.7 years; 95.2% were male. Soccer was the most common injury mechanism (52.4%), followed by athletics and running. The predominant fracture type was Ogden IVb (38.1%). Overweight/obesity was present in 52.4% of the patients, significantly higher than the national benchmarks. An open reduction and internal fixation was performed in 90.5% of the cases, with a mean follow-up of 14.6 months (range: 6–36). Complications occurred in 14.3% overall, all within the overweight/obese group (27.3%). *Conclusions*: This pilot retrospective study suggests a potential link between an elevated BMI and tibial tuberosity avulsion fractures, with overweight/obesity being significantly more prevalent in affected patients than in the general pediatric population. These exploratory findings warrant confirmation in larger, adequately powered studies, and emphasize the importance of weight management and tailored sports activities as potential preventive strategies. An early diagnosis, timely surgical intervention, and adequate rehabilitation are critical for achieving optimal functional recovery.

## 1. Introduction

Tibial tuberosity fractures are rare, comprising less than 1% of epiphyseal injuries and about 3% of proximal tibial fractures, with an incidence of 0.4% to 2.7% among pediatric fractures [1,2,3,4,5]. These injuries most commonly affect adolescents aged 12 to 16 who are approaching skeletal maturity, a phase when the physis becomes less resistant to tensile forces [5,6,7]. A forceful quadriceps contraction with the knee extended or rapid passive knee flexion against a contracted quadriceps are the typical mechanisms of injury [7].

The proximal tibial physis closes in a postero-anterior and craniocaudal manner. Fracture patterns vary depending on the degree of physeal maturity and knee flexion at the time of the injury. Isolated tibial tuberosity fractures usually occur with the knee in extension or flexion less than 30 degrees, while greater flexion is often associated with combined fractures involving the tuberosity and proximal epiphysis [4,8,9]. Predisposing factors, including Osgood–Schlatter disease, osteogenesis imperfecta, patella baja, and an elevated body mass index (BMI), may increase the fracture risk by weakening the tibial tuberosity or elevating mechanical stress [2,3,4,6,7].

**Figure 1:**
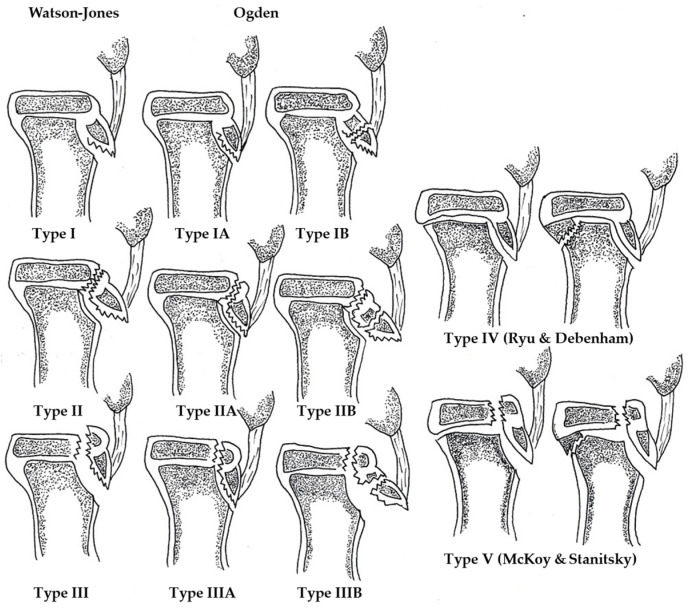
Modified Ogden classification of tibial tuberosity fractures. The schematic illustrates the Watson-Jones classification (types I–III) and its later modifications by Ogden (subtypes A and B), Ryu and Debenham (type IV), and McKoy and Stanitsky (type V). The figure was hand-drawn by the first author based on published depictions.

Classification systems guide treatment decisions for these fractures. Watson-Jones introduced the first radiological classification based on the fracture location and the involvement seen on lateral knee radiographs [10]. Ogden later added subcategories for nondisplaced (type A) and displaced or comminuted (type B) fractures [11]. Subsequent modifications by Ryu, Debenham, and others refined the system [12,13,14]. This classification is illustrated in Figure 1.

More recently, Pandya developed a four-stage classification that incorporates three-dimensional anatomy and physeal closure patterns, enabling advanced diagnostics and early injury detection [15].

Treatment aims to restore the extensor mechanism and, when necessary, the articular surface, while addressing any associated soft-tissue injuries.

While previous studies have primarily focused on age, sex, and sport type, there are limited data on the potential role of an elevated BMI as a risk factor, and the association between the BMI and these injuries remains underexplored.

The present study aimed to (1) describe the epidemiological and anthropometric characteristics of pediatric patients with tibial tuberosity avulsion fractures; (2) analyze the distribution of injury mechanisms and fracture types; and (3) test whether the prevalence of overweight/obesity among cases exceeded national population benchmarks. We hypothesized that the proportion of overweight/obese patients with tibial tuberosity avulsion fractures would be significantly higher than in the general pediatric population. Additionally, the treatment outcomes and complications observed during follow-up are presented descriptively to provide a more complete clinical picture of the included patients.

## 2. Materials and Methods

This retrospective study reviewed the medical records of pediatric and adolescent patients treated for tibial tuberosity avulsion fractures at a single tertiary institution between January 2017 and January 2024. Patients were eligible if they were under 18 years of age, had radiologically confirmed tibial tuberosity avulsion fractures, were treated surgically, and had a minimum follow-up of six months. We included only surgically treated patients to ensure a homogeneous study population with complete operative details and follow-up data, allowing for a reliable assessment of radiographic healing, functional recovery, and potential postoperative complications. A minimum follow-up of six months was provisionally selected by the authors, as this period was considered sufficient to detect clinically relevant outcomes, including late complications such as hardware prominence or growth disturbances. Patients with isolated fractures of the tibial eminence, tibial plateau, or proximal growth plate, or those with less than six months of follow-up, were excluded. Data were collected from the hospital information system and patient records. The demographic variables included age, sex, height, and weight. The height and weight were measured during the preoperative hospital admission as part of a routine clinical assessment and recorded in the medical records. The pubertal stage was not routinely documented in medical records during the study period and could not be assessed retrospectively. Injury-related data included the mechanism of injury, the side affected, the presence of concomitant injuries in the same knee, and the presence of any systemic musculoskeletal conditions. The time from injury to surgery was also recorded.

Surgical reports were reviewed to determine the type of surgical procedure performed, the intraoperative findings, and the duration of surgery. An intraoperative evaluation of the patellar ligament was performed by direct inspection after the exposure of the tibial tuberosity. If disruption or avulsion was present, the ligament was repaired either via transosseous sutures or direct reattachment using non-absorbable sutures, depending on the extent of damage.

The fractures were treated by either closed or open reduction, followed by internal fixation using either 4.0 mm cannulated screws with washers or Kirschner wires. Open reductions were performed via subperiosteal exposure with anatomical repositioning, taking care to avoid damage to the physis.

The radiological evaluation included standard anteroposterior and lateral knee radiographs in all cases. Computed tomography (CT) scans were performed in eleven patients (57.9%), and one patient underwent MRI. The fractures were classified according to the modified Ogden classification. Classification was conducted independently by two orthopedic surgeons using radiographs; disagreements were resolved by consensus. When available, CT and MRI were used to refine the classification and assess the articular involvement. Postoperative radiographs were used to evaluate radiological healing and detect complications.

The anthropometric measurements (height, weight, BMI) were analyzed using the CDC BMI Percentile Calculator for Children and Teens. Patients were categorized as normal weight (<85th percentile) or overweight/obese (≥85th percentile), according to CDC criteria [16]. These proportions were then compared with national prevalence data, including recent adolescent data and annual national surveillance reports [17,18].

The postoperative data included the immobilization time, rehabilitation protocol, knee function at final follow-up, time to hardware removal, and presence of complications or reoperations. The postoperative care followed our institutional protocol: the operated limb was immobilized in an above-knee cast for four weeks, with partial weight-bearing initiated after cast removal. Supervised physiotherapy focusing on range of motion and quadriceps strengthening began thereafter. A return to full sports activities was generally permitted after 4–6 months, depending on the clinical and radiographic recovery.

A statistical analysis was performed using the MedCalc^®^ Statistical Software, version 23.1.3 (MedCalc Software Ltd., Ostend, Belgium; 2025). Categorical variables were summarized as absolute frequencies and percentages, and analyzed using Fisher’s exact test. Cramer’s V was calculated for the effect size. Continuous variables were reported as medians with interquartile ranges and compared using the Mann–Whitney U test. The Hodges–Lehmann estimate was used to report the median difference. For the primary outcome, we applied a one-sample exact binomial test with a one-sided alternative hypothesis (*p* > *p*_0_). Exact one-sided binomial power analyses were performed in R, version 4.4.3 (R Foundation for Statistical Computing, Vienna, Austria). A *p*-value of <0.05 was considered statistically significant.

This study was approved by the institutional ethics committee of University Hospital Centre Osijek (approval number: R 1-398/2025, date of approval: 17 January 2025) and conducted in accordance with the principles of the Declaration of Helsinki.

## 3. Results

**Figure 2:**
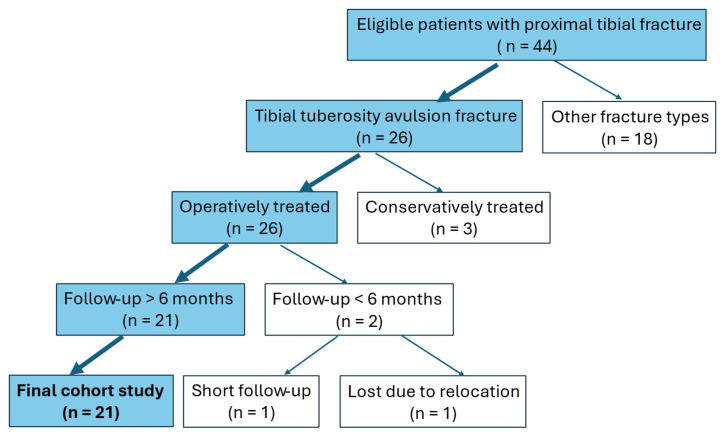
Flow chart showing eligibility screening, exclusion, and final study sample.

This study initially screened 44 pediatric and adolescent patients with proximal tibial injuries who met the general eligibility criteria. Among these, 26 had tibial tuberosity avulsion fractures. Three patients were excluded because they were treated conservatively. Two surgically treated patients were excluded due to insufficient follow-up: one was lost to follow-up because of relocation, and one had a follow-up period shorter than six months. The remaining 21 patients met all the inclusion criteria and were included in the final analysis (Figure 2).

The mean age at the time of injury was 13.71 years (range: 6 years and 4 months to 16 years and 3 months). The majority of the patients were male (20 out of 21, 95.2%). The demographic and anthropometric characteristics of the study sample are presented in Table 1.

The most common mechanism of injury was soccer (11 patients, 52.4%), followed by athletics (4 patients, 19.1%), running (3 patients, 14.3%), and basketball (2 patients, 9.5%) (Table 2).

The youngest patient in our study was a six-year-old girl who sustained the injury while jumping on a trampoline. Apart from obesity (92.7 BMI percentile), her medical records did not reveal any underlying musculoskeletal or metabolic abnormalities.

An anthropometric analysis showed that the average height, expressed as a percentile for age and sex, was 88.6 (IQR: 74.7–98.0), and the average weight was 91.3 (IQR: 76.7–98.0). In this pilot retrospective study, the mean BMI was in the 87.1 percentile (IQR: 60.1–95.8) and we found that more than half of the pediatric patients with tibial tuberosity avulsion fractures (52.4%) were overweight or obese. While differences in weight and the BMI between the normal-weight and overweight/obese groups were expected, they were also statistically significant: the overweight/obese patients were slightly taller (Hodges–Lehmann median difference of 3.4), were significantly heavier (*p* < 0.001; Hodges–Lehmann of 22.3), and had markedly higher BMI percentiles (*p* < 0.001; Hodges–Lehmann of 42.4) (Table 3).

A further analysis revealed that overweight/obese patients were, on average, younger than their normal-weight peers (mean age: 13.7 vs. 14.8 years; median: 13.6 vs. 14.7 years; *p* = 0.26). Regarding sport participation, athletics-related injuries appeared to be more common among overweight/obese patients (27.3% vs. 10.0%), whereas soccer was the leading cause in both groups (45.5% vs. 60.0%) (Table 1 and Table 2).

The radiological evaluation included standard X-rays in all patients. CT scans were performed in thirteen patients (61.9%), and one patient underwent MRI (4.8%). Fractures were classified according to the modified Ogden classification. The most common type was Ogden IVb (eight patients, 38.1%), followed by IIIa (six patients, 28.6%), and Ib and IIIb (two patients, 9.5%). Combined, types III and IV accounted for 81% of all fractures (Table 2). Osgood–Schlatter disease was noted in two patients (9.5%) (Table 1).

**Figure 3:**
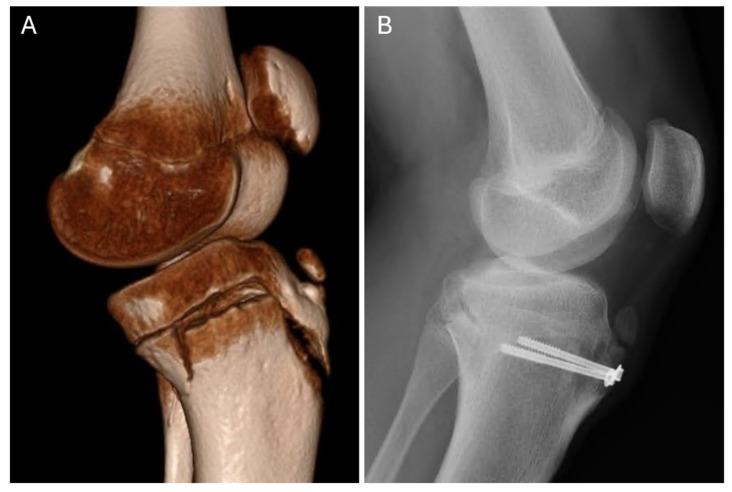
Three-dimensional computed tomography and postoperative radiographs demonstrating an Ogden type IVb tibial tuberosity fracture with residual changes of Osgood–Schlatter disease. (**A**) A 3D-CT reconstruction of a type IVb tibial tuberosity fracture with residual Osgood–Schlatter disease. (**B**) Postoperative radiograph taken 10 weeks after osteosynthesis with two cancellous screws and washers, showing a healed fracture.

**Figure 4:**
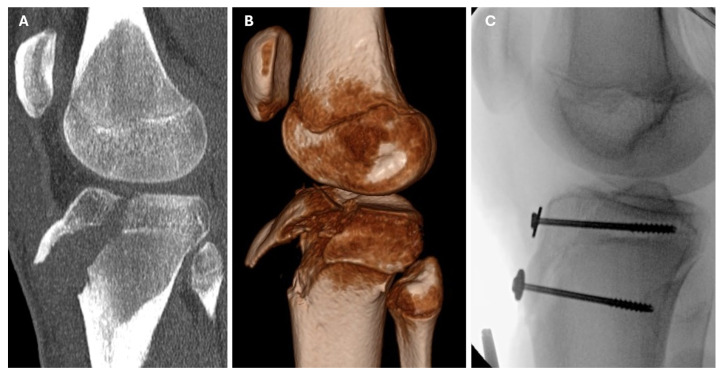
CT, 3D-CT, and intraoperative radiographic views of a type IIIb tibial tuberosity fracture. (**A**) Sagittal CT scan. (**B**) A 3D-CT reconstruction view of the fracture. (**C**) Intraoperative imaging following an open reduction and internal fixation using two cancellous screws and washers.

**Figure 5:**
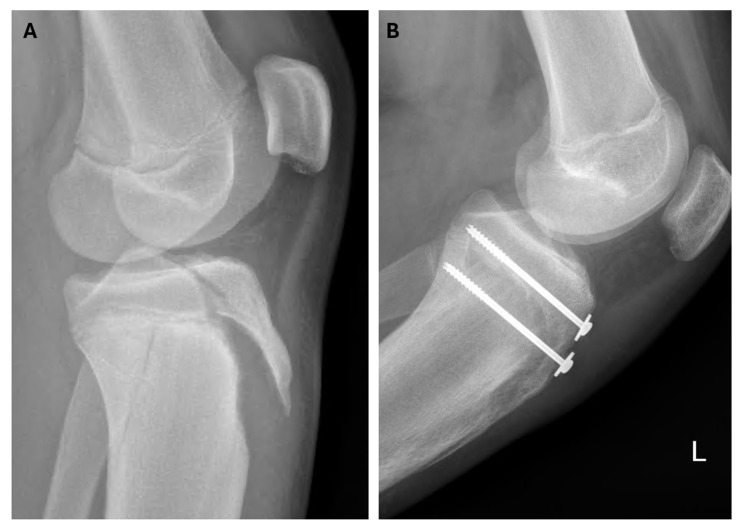
Radiographic images of a type V tibial tuberosity fracture. (**A**) Preoperative radiograph of a type V tibial tuberosity fracture with extension of the fracture line into the tibial metaphysis. (**B**) Postoperative radiograph demonstrating a healed fracture 7 weeks after surgery. L indicates left side.

All the patients underwent surgical treatment. A closed reduction and internal fixation (CRIF) was performed in 2 cases (9.5%), while an open reduction and internal fixation (ORIF) was used in the remaining 19 patients (90.5%). Kirschner wires were used in one case, while 4.0 mm cannulated screws with washers were used in the others. Figure 3, Figure 4 and Figure 5 show radiological and intraoperative images of patients from our study sample.

The intraoperative findings included a patellar ligament injury in 10 patients (47.6%), with 9 of these in the normal-weight group (*p* < 0.001). These injuries were treated by primary repair or transosseous reattachment. Additional soft-tissue injuries included quadriceps tendon tears, retinacular damage, tibialis anterior muscle involvement, and, in one case, skin avulsion.

Postoperative complications were observed in three patients (14.3%). While not initially hypothesized, it is notable that all complications occurred in overweight patients (27.3%), suggesting a potential association that warrants further investigation. Early complications included one superficial wound infection and one case of wound dehiscence. A late complication involved intra-articular screw prominence requiring reoperation (Table 4).

The median time from admission to surgery was 5.8 h (IQR: 3.1–15.8), and the median surgical duration was 70 min (IQR: 50.0–95.0). The patients were immobilized for a median of 37 days (IQR: 31–40), with radiological healing occurring at a median of 38 days (IQR: 34.0–44.0). All the patients regained their pre-injury knee function and returned to their previous activities. The hardware was removed after a median of 244 days (IQR: 208.0–291.0), as per institutional protocol. The perioperative and postoperative time intervals were not compared between the BMI groups due to the small sample size and uneven distribution (Table 5).

## 4. Discussion

Tibial tuberosity fractures, although rare, represent a relevant concern in pediatric and adolescent orthopedics. Our findings confirm that these injuries typically occur in adolescent boys aged 12–16, corresponding with a period of increased mechanical vulnerability due to physeal maturation. In our study, the average patient age was 13.71 years, which aligns with the existing literature [1,2,3,4,5]. Notably, our youngest patient was a six-year-old girl who sustained the injury during trampoline activity, without other known risk factors beyond a BMI in the 92.7 percentile.

The physeal anatomy of the tibial tuberosity is a key factor in injury susceptibility. As described by Ogden, ossification progresses from posteromedial to anterolateral, with fibrocartilage gradually replaced by hypertrophic columnar cartilage, which is less resistant to tensile forces, making the area more vulnerable to avulsion fractures as the ossification center nears fusion with the metaphysis. This transition, combined with strong quadriceps contractions during physical activity, increases the likelihood of avulsion injuries [11,14].

The mechanisms leading to these fractures are well established in the literature [7]. This explains the prevalence of these injuries in adolescents participating in sports that involve explosive movements [1]. In Europe, soccer is often the dominant organized sport among male adolescents, whereas studies from North America have reported basketball as the predominant sport associated with these fractures [1,4,5,19,20,21,22]. Such differences underscore the importance of considering regional patterns of youth sports engagement when interpreting epidemiological findings. A considerable proportion of patients in our study were classified as overweight or obese, with significantly higher body mass indices compared to their normal-weight peers. This prevalence was substantially higher than national reference data. Recent adolescent data reported an overall prevalence of 15.4% and 25.6% among boys, while national surveillance data indicate a prevalence of approximately 20% across school-age children [17,18]. This discrepancy suggests that an elevated BMI may contribute to the risk of tibial tuberosity avulsion fractures, although causality cannot be established from our design. While an increased body mass is an intuitive risk factor, it may interact with sport-specific loading patterns in meaningful ways. According to Nikander et al., soccer is categorized as an “odd-impact” activity characterized by rapid changes in direction, accelerations, and decelerations that generate high strain rates and multidirectional loading on the tibia, resulting in an increased cortical thickness and a larger cross-sectional geometry of the tibia due to periosteal expansion [23,24]. The change in cartilage composition, together with mechanical stimuli and the increased joint reaction forces associated with a higher body mass, may further concentrate stress at specific sites, creating focal areas of vulnerability at the apophysis of the tibial tuberosity. This interaction between an elevated BMI and odd-impact loading could contribute to the observed injury pattern in our overweight and obese patients. Our results align with previous reports of Haber and Reyes potentially identifying excess body weight [3,6]. In addition to mechanical loading, other factors may help explain the link between a higher BMI and tibial tuberosity avulsion fractures. Adolescents with a higher BMI often have a lower physical fitness and less-efficient movement patterns, which can reduce coordination during sport-specific activities and increase the injury risk [25]. Although we did not collect data on how long or how often the participants played sports, these factors, along with the previously described mechanical and physeal vulnerabilities, may all contribute to the multifactorial nature of the injury risk in this group.

Based on our findings and the increasing trend in the fracture incidence observed at our hospital in recent years, and despite the declining birth rate, we are inclined to attribute this rise, at least in part, to the growing prevalence of an elevated BMI among adolescents in our population [26,27]. While the small sample size warrants caution in interpreting these results, exact one-sided binomial power analyses indicated that this study was adequately powered to detect a clinically meaningful difference, despite the limited N. Our study supports existing claims in the literature and reveals a clear trend that could be validated in future studies with larger sample sizes. Further research should aim to determine the specific BMI percentile threshold that constitutes a significant risk for this injury.

The male predominance observed in our study (95.2%) aligns with the existing literature, which attributes this disparity to both hormonal differences and gender-based variations in sports participation [1,4,19]. Males generally experience delayed skeletal maturation, which shifts the period during which the tibial tuberosity is vulnerable to injury to a later stage of adolescence, when they are more likely to participate in high-intensity competitive sports. In contrast, girls often reduce their participation in vigorous physical activities at a younger age, which may limit their exposure to the mechanical loads associated with these injuries during the period of physeal vulnerability [1]. Furthermore, the greater muscle mass in male adolescents, combined with the delayed ossification of both the tibial tuberosity and the metaphysis, may generate higher tensile forces on the tibial tuberosity. These biomechanical factors, along with behavioral differences, contribute to the higher incidence of tibial tuberosity fractures in males.

In our study, the most common fracture type according to the modified Ogden classification was Ogden type IVb (38.1%), followed by type IIIa (28.6%). Overall, types III and IV comprised 81% of all the fractures. However, no statistically significant associations were found between the fracture type and the injury mechanism, body weight, or age. According to Haber et al., in a study of 236 patients, the most common fracture types were Ogden type III (41%) and type I (29%). They also found a lower average age in patients with lower Ogden types and an association between type II and type III fractures and an elevated BMI. Osgood–Schlatter disease, as a risk factor, was generally associated with lower Ogden fracture types [3]. In our study, we identified two patients with Osgood–Schlatter disease.

Due to the rarity of tibial tuberosity fractures, various treatment approaches are discussed in the literature. A conservative treatment with immobilization can be effective for nondisplaced or minimally displaced fractures (types IA, IB, and IIA), provided the extensor mechanism remains intact, as described in the literature [2,3,5,6,14]. Additionally, even type IV fractures treated conservatively have been reported to achieve full knee function after treatment [28].

According to Pretell-Mazzini et al., 88% of patients with tibial tuberosity avulsion fractures require surgery, with 98% undergoing an ORIF [4]. An ORIF is the gold standard for displaced, comminuted, and articular fractures (types IIB, IIIA, IIIB, and IV). The main goals of treatment are to repair the extensor mechanism, reconstruct the articular surface, and address any associated injuries. Surgical methods typically include fixation with screws and Kirschner wires [3,4,5,6,7,8,19,20].

The surgical treatment approach in our study followed these established protocols. A CRIF was employed for two fractures (9.5%), while an ORIF was used for the remaining fractures (90.5%). Among the ORIF procedures, most fractures were treated with 4.0 mm cannulated screws and washers, with periosteal repositioning to ensure precise reduction. In line with Rodriguez et al., we recommend periosteal suturing to promote proper healing and facilitate earlier rehabilitation [8].

Additionally, the high incidence of associated patellar ligament injuries in our study (47.6%) underscores the importance of a thorough intraoperative evaluation to properly address and treat injuries to the extensor apparatus, preventing potential long-term functional deficits and ensuring the early functional restoration of the knee. This injury was significantly more frequent among patients with a normal BMI compared to their overweight peers (9 vs. 1; *p* < 0.001). Patellar ligament and quadriceps tendon injuries were managed with primary repair or transosseous reattachment. All the patients achieved full functional recovery with a symmetric range of motion comparable to the contralateral limb and returned to pre-injury activity levels. According to the literature, 98% of surgically treated patients regained full range of motion within 22 weeks and returned to sports activities after 29 weeks [4,8].

Complications were observed in 14.3% of the patients, all of whom were obese. When analyzed within the BMI subgroups, complications were present in 27.3% of the overweight or obese patients. These included minor wound issues and one case of intra-articular screw prominence requiring reoperation, and this was consistent with the findings in the literature [3,4]. In a systematic review by Pretell-Mazzini et al., the overall complication rate was reported to be 28%. The most common complications leading to the removal of osteosynthesis material were bursitis (56%) and prominence of the material, which caused palpable tenderness or numbness of the tibial tuberosity and pain during squatting [3,4].

Serious neurovascular complications, such as acute compartment syndrome from injury to branches of the anterior recurrent tibial artery, occurred in 2–21% of cases. Frey reported a 21% incidence in a study of 19 adolescents with tibial tuberosity fractures, while Haber found a 2% incidence, attributed to prophylactic fasciotomy [3,5]. In our study, no cases of acute compartment syndrome were observed, likely due to the small sample size and short time from hospital admission to surgery.

### Study Limitations

This study has several limitations. The small sample size limits its generalizability and precludes causal inference. Nevertheless, the effect size for the primary outcome was large, and the observed prevalence of overweight/obesity (52.4%) significantly exceeded the national benchmarks. Exact one-sided binomial power analyses in R confirmed that, despite the limited cohort size, the post hoc power remained high across the reference prevalences (97.6% for *p*_0_ = 0.154, 93.8% for *p*_0_ = 0.200, and 74.5% for *p*_0_ = 0.256). A priori calculations further indicated that 12–20 patients would be required for 80% power and 13–29 would be needed for 90% power, depending on the benchmark used. These results suggest that the primary finding is adequately powered, while also providing clear numerical guidance for future research design. The retrospective design and reliance on existing documentation may have led to the underreporting of certain clinical variables. There was no control group for comparison with the general pediatric population or with conservatively treated patients. Only surgically treated patients with a minimum follow-up of six months were included to ensure a homogeneous study population and a reliable assessment of the functional and radiographic outcomes; however, this approach may limit the applicability to the broader patient population. Accordingly, the present work should be considered a pilot retrospective study, and the findings interpreted as exploratory, indicating possible associations rather than establishing causation. Finally, the follow-up period of a minimum of six months may be insufficient to detect late complications, such as growth disturbances or genu recurvatum. Further studies with larger sample sizes are needed to confirm these preliminary observations.

## 5. Conclusions

Tibial tuberosity avulsion fractures are rare injuries most commonly seen in adolescent males during high-intensity physical activity. This pilot retrospective study suggests a potential link between an elevated BMI and these fractures, with overweight/obesity being significantly more prevalent in affected patients than in the general pediatric population. These exploratory findings warrant confirmation in larger, adequately powered studies. Nevertheless, they highlight the need for an increased awareness of obesity as a potential contributing factor in sports-related injuries and emphasize the importance of early recognition, timely surgical intervention, and appropriate rehabilitation. Surgical treatment yielded excellent functional outcomes in all patients, underscoring its role as the standard of care in this patient population.

## Figures and Tables

**Table 1:** Demographic and anthropometric characteristics of patients *.

Patient	Sex	Age	Side	Osgood–Schlatter Disease	BMI (kg/m^2^)	BMI Percentile
1	M	16	R	-	24.8	87.1
2	M	12	L	-	18.0	44.3
3	M	14	L	-	32.7	98.9
4	M	13	L	-	24.8	93.2
5	M	13	L	-	27.2	96.8
6	M	15	L	-	23.2	80
7	M	14	R	-	33.3	98.6
8	F	6	L	-	18.4	92.7
9	M	13	L	Yes	20.3	72
10	M	14	L	-	18.3	26
11	M	12	R	-	23.8	92.5
12	M	16	L	-	20.1	40.5
13	M	15	L	-	33.8	99.1
14	M	14	L	-	20.8	67.4
15	M	14	L	Yes	19.3	46.6
16	M	13	L	-	25.8	95.8
17	M	14	L	-	21.1	69.1
18	M	15	L	-	20.8	60.1
19	M	16	L	-	24.9	87.8
20	M	16	R	-	20.3	44.2
21	M	13	R	-	32.4	98

* Abbreviations: M—male; F—female; R—right; L—left; BMI—body mass index.

**Table 2:** Clinical and fracture characteristics of patients *.

Patient Number	Sport Causing Injury	Additional Imaging	Modified Ogden Classification	Treatment Method	Fixation Type	Associated Injuries	Complications	Follow-Up (Months)
1	Soccer	MRI, CT	Ib	ORIF	screws	patellar ligament	wound dehiscence	10.8
2	Athletics	-	IVb	ORIF	K wires	patellar ligament, tibialis anterior muscle	-	10.0
3	Soccer	-	IIIa	ORIF	screws	patellar ligament	-	6.8
4	Soccer	-	IIIb	ORIF	screws	-	-	11.6
5	Handball	CT	IIIb	ORIF	screws	quadriceps tendon	intra-articular screw prominence	10.7
6	Running	-	IIIa	ORIF	screws	patellar ligament	-	8.7
7	Running	CT	IIIa	ORIF	screws	retinacula	-	18.7
8	Athletics	CT	IVa	CRIF	K wires	-	-	18.4
9	Soccer	CT	IVb	ORIF	screws	patellar ligament	-	7.8
10	Soccer	-	IIIb	ORIF	screws	patellar ligament	-	9.2
11	Athletics	CT	Ib	ORIF	screws	-	-	8.5
12	Basketball	CT	IVb	ORIF	screws	retinacula, patellar luxation, fibular head fracture	-	7.3
13	Athletics	-	IVb	CRIF	screws	-	-	29.9
14	Soccer	CT	IIIa	ORIF	screws	quadriceps tendon	-	7.4
15	Soccer	CT	IIb	ORIF	screws	patellar ligament	-	10.0
16	Soccer	-	IVb	ORIF	screws	-	-	10.2
17	Running	CT	IVb	ORIF	screws	patellar ligament	-	7.2
18	Soccer	CT	IIIa	ORIF	screws	patellar ligament	-	9.7
19	Basketball	-	IVb	ORIF	screws	fibular head fracture, skin avulsion	superficial skin infection	7.9
20	Soccer	CT	IVb	ORIF	screws	retinacula	-	7.2
21	Soccer	CT	V	ORIF	screws	patellar ligament	-	7.6

* Abbreviations: MRI—magnetic resonance imaging; CT—computed tomography; ORIF—open reduction and internal fixation; CRIF—closed reduction and internal fixation; K—Kirschner wire.

**Table 3:** Descriptive analysis of height, weight, and BMI percentiles with statistical comparison.

Variable	Me (IQR)	Min–Max	Me (IQR)		*p* **	Hodges–Lehmann Median Difference
	Total		Normal <85th Percentile	Overweight ≥85th Percentile		95%CI
Height percentile	88.6 (74.7 to 98.0)	25.0 to 99.9	85.9 (67.5 to 91.8)	89.3 (77.3 to 98.9)	0.46	3.4 (−41.1 to 73.9)
Weight percentile	91.3 (76.7 to 98.0)	34.6 to 99.7	75.7 (61.3 to 82.1)	98.0 (95.4 to 99.5)	<0.001	22.3 (1.5 to 64.9)
BMI percentile	87.1 (60.1 to 95.8)	26.0 to 99.1	53.4 (44.2 to 68.7)	95.8 (92.6 to 98.3)	<0.001	42.4 (6.0 to 52.5)

Abbreviations: Me—median; IQR—interquartile range; Min—minimum; Max—maximum; *p*—probability; BMI—body mass index; ** Mann–Whitney test; CI—confidence interval.

**Table 4:** Association of categorical variables with BMI categories: descriptive statistics and effect sizes *.

Variable	Category	n (%)			*p* **	Cramer’s V ^†^
		Total	Normal <85th Percentile	Overweight ≥85th Percentile		
Sex	M	20 (95.2)	10 (100.0)	10 (90.9)	>0.99	0.218
	F	1 (4.8)	0	1 (9.1)		
Modified Ogden Classification	Ib	2 (9.5)	1 (10.0)	1 (9.1)	<0.99	0.282
	IIb	1 (4.8)	1 (10.0)	0		
	IIIa	6 (28.6)	3 (30.0)	3 (27.3)		
	IIIb	2 (9.5)	1 (10.0)	1 (9.1)		
	IVa	1 (4.8)	1 (10.0)	0		
	IVb	8 (38.1)	4 (40.0)	4 (36.4)		
	V	1 (4.8)	0	1 (9.1)		
Side	R	5 (23.8)	2 (20.0)	3 (27.3)	>0.99	0.117
	L	16 (76.2)	8 (80.0)	8 (72.7)		
Associated Injuries	none	6 (28.6)	0	6 (54.5)	0.01	0.612
	patellar ligament	10 (47.6)	9 (90.0)	1 (9.1)	<0.001	0.788
	tibialis anterior muscle	1 (4.8)	1 (10.0)	0	>0.99	0.218
	quadriceps tendon	2 (9.5)	0	2 (18.2)	0.48	0.343
	retinacula	3 (14.3)	1 (10.0)	2 (18.2)	>0.99	0.148
	patellar luxation	1 (4.8)	0	1 (9.1)	0.47	0.248
	fibular head fracture	2 (9.5)	0	2 (18.2)	0.48	0.343
	skin avulsion	1 (4.8)	0	1 (9.1)	>0.99	0.218
Complications	yes	3 (14.3)	0	3 (27.3)	0.21	0.411
	no	18 (85.7)	10 (100.0)	7 (72.7)		

* Abbreviations: M—male; F—female; R—right; L—left; n—number; *p*—probability; ** Fisher’s exact test; ^†^ small effect size, 0.10; medium effect size, 0.30; large effect size, 0.50; the percentage of shared variance was calculated as the square × 100.

**Table 5:** Descriptive statistics for perioperative and postoperative time intervals *.

Variable	Me (IQR)	Min–Max
Time until surgery (h)	5.8 (3.1 to 15.8)	1.8–73.5
Duration of surgery (min)	70 (50.0–95.0)	30–120
Immobilization time (days)	37 (31 to 40)	20–46
Radiographic healing time (days)	38 (34–44)	20–65
Hardware removal (days)	244 (208–291)	37–556

* Abbreviations: Me—median; IQR—interquartile range; Min—minimum, Max—maximum.

## Data Availability

The data supporting the findings of this study are available from the corresponding author upon reasonable request.

## Abbreviations

The following abbreviations are used in this manuscript:

BMI	Body Mass Index
CDC	Centers for Disease Control and Prevention
CRIF	Closed Reduction and Internal Fixation
CT	Computed Tomography
IQR	Interquartile Range
MRI	Magnetic Resonance Imaging
ORIF	Open Reduction and Internal Fixation

## References

[1] Brey J.M., Conoley J., Canale S.T., Beaty J.H., Warner W.C., Kelly D.M., Sawyer J.R. (2012). Tibial tuberosity fractures in adolescents: Is a posterior metaphyseal fracture component a predictor of complications?. J. Pediatr. Orthop..

[2] Bolesta M.J., Fitch R.D. (1986). Tibial tubercle avulsions. J. Pediatr. Orthop..

[3] Haber D.B., Tepolt F.A., McClincy M.P., Hussain Z.B., Kalish L.A., Kocher M.S. (2021). Tibial tubercle fractures in children and adolescents: A large retrospective case series. J. Pediatr. Orthop. B.

[4] Pretell-Mazzini J., Kelly D.M., Sawyer J.R., Esteban E.M., Spence D.D., Warner W.C., Beaty J.H. (2016). Outcomes and Complications of Tibial Tubercle Fractures in Pediatric Patients: A Systematic Review of the Literature. J. Pediatr. Orthop..

[5] Frey S., Hosalkar H., Cameron D.B., Heath A., David Horn B., Ganley T.J. (2008). Tibial tuberosity fractures in adolescents. J. Child. Orthop..

[6] Reyes C.D., Wu W., Pandya N.K. (2023). Adolescent Tibial Tubercle Fracture: Review of Outcomes and Complications. Curr. Rev. Musculoskelet. Med..

[7] Hamilton S.W., Gibson P.H. (2006). Simultaneous bilateral avulsion fractures of the tibial tuberosity in adolescence: A case report and review of over 50 years of literature. Knee.

[8] Rodriguez I., Sepúlveda M., Birrer E., Tuca M.J. (2020). Fracture of the anterior tibial tuberosity in children. EFORT Open Rev..

[9] Jakoi A., Freidl M., Old A., Javandel M., Tom J., Realyvasquez J. (2012). Tibial tubercle avulsion fractures in adolescent basketball players. Orthopedics.

[10] Watson-Jones R. (1955). Fractures and Joint Injuries.

[11] Ogden J.A., Tross R.B., Murphy M.J. (1980). Fractures of the tibial tuberosity in adolescents. J. Bone Jt. Surg. Am..

[12] Ryu R.K., Debenham J.O. (1985). An unusual avulsion fracture of the proximal tibial epiphysis: Case report and proposed addition to the Watson-Jones classification. Clin. Orthop. Relat. Res..

[13] Frankl U., Wasilewski S.A., Healy W.L. (1990). Avulsion fracture of the tibial tubercle with avulsion of the patellar ligament. Report of two cases. J. Bone Joint Surg. Am..

[14] McKoy B.E., Stanitski C.L. (2003). Acute tibial tubercle avulsion fractures. Orthop. Clin. N. Am..

[15] Pandya N.K., Edmonds E.W., Roocroft J.H., Mubarak S.J. (2012). Tibial tubercle fractures: Complications, classification, and the need for intra-articular assessment. J. Pediatr. Orthop..

[16] CDC BMI Percentile Calculator for Children and Teens (2023). Centers for Disease Control and Prevention. https://www.cdc.gov/bmi/child-teen-calculator/widget.html.

[17] Hrvatski Zavod za Javno Zdravstvo (2024). Hrvatski Zdravstveno-Statistički Ljetopis za 2023. https://www.hzjz.hr/hrvatski-zdravstveno-statisticki-ljetopis/hrvatski-zdravstveno-statisticki-ljetopis-za-2023-g-tablicni-podaci/.

[18] Matana A., Krajinović H. (2024). Prevalence of Overweight and Obesity and Association with Risk Factors in Secondary School Children in Croatia. Children.

[19] Formiconi F., D’Amato R.D., Voto A., Panuccio E., Memeo A. (2020). Outcomes of surgical treatment of the tibial tuberosity fractures in skeletally immature patients: An update. Eur. J. Orthop. Surg. Traumatol..

[20] Cole W.W., Brown S.M., Vopat B., Heard W.M.R., Mulcahey M.K. (2020). Epidemiology, Diagnosis, and Management of Tibial Tubercle Avulsion Fractures in Adolescents. JBJS Rev..

[21] Mubarak S.J., Kim J.R., Edmonds E.W., Pring M.E., Bastrom T.P. (2009). Classification of proximal tibial fractures in children. J. Child. Orthop..

[22] Ares O., Seijas R., Cugat R., Alvarez P., Aguirre M., Catala J. (2011). Treatment of fractures of the tibial tuberosity in adolescent soccer players. Acta Orthop. Belg..

[23] Nikander R., Kannus P., Rantalainen T., Uusi-Rasi K., Heinonen A., Sievänen H. (2010). Cross-sectional geometry of weight-bearing tibia in female athletes subjected to different exercise loadings. Osteoporos. Int..

[24] O’Leary T.J., Rice H.M., Greeves J.P. (2021). Biomechanical Basis of Predicting and Preventing Lower Limb Stress Fractures During Arduous Training. Curr. Osteoporos. Rep..

[25] Barros W.M.A., da Silva K.G., Silva R.K.P., Souza A.P.D.S., da Silva A.B.J., Silva M.R.M., Fernandes M.S.S., de Souza S.L., Souza V.O.N. (2022). Effects of Overweight/Obesity on Motor Performance in Children: A Systematic Review. Front. Endocrinol..

[26] Rodin U., Cerovecki I., Jezdic D. (2024). Demographic Trends in Croatia in 2023. Hrvatski Zavod za Javno Zdravstvo. https://www.hzjz.hr/wp-content/uploads/2024/10/HZJZ_-_prirodno_kretanje_2023._g..pdf.

[27] Musić Milanović S., Lang Morović M., Križan H. (2021). European Childhood Obesity Initiative. Croatia 2018/2019 (CroCOSI).

[28] Checa Betegón P., Arvinius C., Cabadas González M.I., Martínez García A., Del Pozo Martín R., Marco Martínez F. (2019). Management of pediatric tibial tubercle fractures: Is surgical treatment really necessary?. Eur. J. Orthop. Surg. Traumatol..

